# Use of Semantic Information to Interpret Thematic Information for Real-Time Sentence Comprehension in an SOV Language

**DOI:** 10.1371/journal.pone.0056106

**Published:** 2013-02-08

**Authors:** Satoru Yokoyama, Kei Takahashi, Ryuta Kawashima

**Affiliations:** 1 Institute of Development, Aging, and Cancer, Tohoku University, Sendai City, Miyagi prefecture, Japan; 2 Graduate School of International Cultural Studies, Tohoku University, Sendai City, Miyagi prefecture, Japan; University of Leicester, United Kingdom

## Abstract

Recently, sentence comprehension in languages other than European languages has been investigated from a cross-linguistic perspective. In this paper, we examine whether and how animacy-related semantic information is used for real-time sentence comprehension in a SOV word order language (i.e., Japanese). Twenty-three Japanese native speakers participated in this study. They read semantically reversible and non-reversible sentences with canonical word order, and those with scrambled word order. In our results, the second argument position in reversible sentences took longer to read than that in non-reversible sentences, indicating that animacy information is used in second argument processing. In contrast, for the predicate position, there was no difference in reading times, suggesting that animacy information is NOT used in the predicate position. These results are discussed using the sentence comprehension models of an SOV word order language.

## Introduction

Recently, sentence comprehension in languages other than European languages has been investigated from a cross-linguistic perspective. In particular, while a large number of studies have already examined the English language so far, the number of studies focusing on the Japanese language, which has many characteristics that differ from the English language, has been increasing [1]–[9]. Between English and Japanese, there are mainly two points related to sentence comprehension that differ: one is the canonical word order (English: SVO; Japanese: SOV), the other is the case marking system (English: word order; Japanese: case particles). Such differences have been assumed to reflect different processing strategies during sentence comprehension between these two languages [10], [11]. However, the number of studies of sentence comprehension in SOV languages, including the Japanese language, remains small, suggesting that the mechanism of sentence comprehension in SOV languages is still unclear. Hence, to totally cover the human language processing mechanism, it is necessary to investigate the sentence comprehension mechanism in SOV languages.

So far, to our knowledge, there are only two models to explain simplex sentence comprehension in the Japanese language: that of Chujo (1983) [12] and that of Yokoyama et al. (2012) [11]. Chujo's Japanese simplex sentence comprehension model proposes that the word order of arguments, the case particle of arguments, and the relationship between arguments and predicate are all used to comprehend a simplex sentence. When the first noun is input, word order information is used. For example, when *John* is input, this noun is interpreted as an actor, as described above. When a case particle follows the first noun, case particle information is used. Since case particle information is superior to the use of word order information, the argument interpretation previously constructed by word order information is revised. For example, when *John-o* (o is an accusative case particle in Japanese) is input, firstly *John* is interpreted as an actor by word order information. But, when *o* is input, *John* is re-interpreted as an undergoer. Finally, when a predicate is input, if there is a discrepancy between the previously constructed argument interpretation and one constructed by the relationship between argument and predicate, the latter information is used and the argument interpretation is revised. For example, when *John-o Mary-ga Hometa* (Mary praised John) is input, since *Hometa* requires a human actor and any undergoer, and *John-o* and *Mary-ga* fulfill the requirement, no revision is necessary and sentence comprehension is completed.

Yokoyama et al.'s model is based on recent works examining previous psycholinguistic and neurolinguistic studies of Japanese sentence comprehension, and is called the partial incremental argument interpretation (PIAI) model. It is like an extended version of Chujo's model, adding some newly obtained findings from recent studies. Basically, in this model, the use of the above three sources of information is the same, but the order of priority and the timing of the use differ from those in Chujo's model. In the PIAI model, if there is a case particle, the case particle information is preferentially used. If there is no case particle, the animacy information of the argument is used. If neither case particle nor animacy information is used, word order information is used. When these kinds of information are used, no revision is required, which differs from Chujo's model. For example, when *John-o* is input, this model predicts that no revision is necessary to re-interpret it as an undergoer rather than an actor. Instead, this model predicts that the first argument is not immediately processed. When both the first and second arguments are input, they are interpreted by using the above three kinds of information. For example, when *John-o Mary-ga* is input, since case particles exist, *John* and *Mary* are interpreted as an undergoer and an actor, respectively. If there are no case particles, for example, when *John pen* is input, by using animacy information, *John* is interpreted as an actor, because it is an animate noun, and *pen* as an undergoer, because it is an inanimate noun. In Chujo's model, semantic information is assumed to be used at the time of predicate processing, while the PIAI model assumes that semantic information is used during argument processing, before predicate processing. In addition, in the latter model, the treatment of the *ni* case particle, which is a dative case particle, differs from that in Chujo's model. While Chujo's model did not mention how *ni* is processed, the PIAI model predicts that a *ni*-marked argument is not immediately processed but rather at the time of predicate processing, differently from the nominative case particle *ga* and the accusative case particle *o*.

However, so far, while the use of animacy information has been confirmed in the off-line questionnaire environment [13], there is no evidence supporting the assumption that animacy information is used for an incremental interpretation of arguments before inputting the predicate in the timeline of a real-time sentence comprehension situation. Regarding the use of animacy information, it is well-known that semantically reversible sentences are more difficult to comprehend than the corresponding non-reversible sentences, due to the animacy information of the arguments [13]–[17]. This difficulty may be caused by the multiple possible interpretations of ‘who does what to whom’ information in the thematic role assignment of the subject and object in reversible sentences in comparison to non-reversible sentences. For example, there are at least two semantic possibilities for thematic role assignment in reversible sentences such as *the boy praised the girl*. One is that the actor is *the boy* and the undergoer is *the girl*. Another is that the actor is *the girl* and the undergoer is *the boy*. In contrast, in non-reversible sentences such as *the boy touched the table*, it is impossible to interpret *the table* as the actor in the thematic role assignment. Hence, since reversible sentence comprehension places less of a constraint on the thematic role assignment of the subject and the object than the corresponding non-reversible one, it is easier to understand non-reversible sentences than reversible sentences.

One recent study regarding reversible sentence comprehension was done by Ferreira [13], and it compared English reversible sentence comprehension with that of non-reversible sentences. In this study, reversible passive sentences (e.g., the dog was bitten by the fox) were misinterpreted by English native speakers. Participants in the study were asked to listen to a stimulus sentence and, afterwards, answer a question regarding who does what to whom, where, and when with regard to the presented stimulus sentence. Unfortunately, it is hard to indicate when participants show difficulty in real-time reversible sentence comprehension.

Furthermore, to our knowledge, there have been only a few studies examining reversible sentence comprehension in the Japanese language. Itoh et al. found that Japanese native speakers used animacy information in argument interpretation as well as case particles [18]. For example, in Japanese, it is possible to drop a case particle, as in *Taro Keeki Tabetayo* (Taro ate the cake). Itoh et al. reported that Japanese native speakers interpreted Taro as the actor and *Keeki* as the undergoer for comprehension of *Keeki Taro Tabetayo*, suggesting that Japanese native speakers used the animacy information of *Taro* and *Keeki* to process these argument interpretations in a no-case particle environment. This finding leads us to postulate that reversibility affects Japanese sentence comprehension as well as English sentence comprehension. A Japanese sentence comprehension model such as Yokoyama et al. (2012) [18] is based on this finding in terms of the incremental process of arguments before inputting a predicate or verb. However, since that study used the off-line questionnaire method, it is still unclear whether animacy information is used for real-time processing of reversible sentences in the Japanese language or not.

Regarding real-time sentence comprehension, there exists only one study examining reversible sentence comprehension in the Japanese language. Suzuki used a self-paced listening task and compared reaction times for each sentence position of the subject, object, and verb in semantically reversible sentences with canonical word order (hereafter, reversible canonical sentences) such as *Inu-ga Buta-o Tatakimashita* (the dog hit the pig), reversible sentences with scrambled word order (hereafter, reversible scrambled sentences) such as *Buta-o Inu-ga Tatakimashita*, non-reversible canonical sentences such as *Kitsune-ga Ichigo-o Tabemashita* (the fox ate the strawberry), and non-reversible scrambled sentences such as *Ichigo-o Kitsune-ga Tabemashita* [19]. That study reported that a semantic reversibility effect was observed at both the second argument position and the verb position. However, since actually used words were critically different between reversible and non-reversible sentences, at least the main effect of the object (e.g., *Buta*: the pig, and *Ichigo*: the strawberry) in reversibility is not enough to test the reversibility effect. In order to indicate that there is a reversibility effect, it is necessary to test the interaction of reversibility×word order in his study. Unfortunately, he found no interaction of reversibility×word order in all positions. This suggests that in a SOV language such as Japanese, reversibility presents no real difficulty for real-time sentence comprehension, which conflicts with the findings of Itoh et al. (1993) [18].

One possible reason why there is a discrepancy between the findings of Itoh et al. (1993) [18] and those from Suzuki (2008) [19] is the experimental method used in Suzuki (2008) [19]. The most plausible reason we can think of is that the self-paced “listening” task used in Suzuki (2008) [19] is not natural enough to see the reversibility effect in real-time sentence comprehension. Although there is, as Suzuki (2008) [19] stated, a previous study suggesting that a self-paced “listening” task shows similar results to eye-tracking experiments for real-time sentence comprehension, the speed of the listening process cannot be controlled in actual daily life, since speakers control the speed of the utterances. In contrast, a self-paced reading task, which has often been used in real-time sentence comprehension studies, is more natural than the self-paced listening task, since the reading process can usually be controlled by the reader. Hence, it is conceivable that a self-paced “listening” task could cause problems when examining real-time reversible sentence comprehension.

Consequently, the purpose of the current study is to examine whether animacy information is used for real-time sentence comprehension in the Japanese language or not. To this end, we used a self-paced “reading” task, which is more natural than a self-paced listening task, and Japanese reversible canonical, reversible scrambled, non-reversible canonical, and non-reversible scrambled sentences as stimuli. By using this task and these stimuli, we tested the following research question derived from previous simplex sentence comprehension models: Is animacy information used during real-time sentence comprehension or not? Chujo's model predicts that semantic information such as animacy information is used in the predicate, since this model proposes that when a predicate is input, if there is a discrepancy between the previously constructed argument interpretation and one constructed by the relationship between argument and predicate, the latter information is used and the argument interpretation is revised. Additionally, this model does not propose that such semantic information is used during argument processing before input of the predicate. Hence, Chujo's model predicts that animacy information is not used in argument processing, but in predicate processing. In contrast, the PIAI model proposes that, in the second argument, the thematic information process begins by using case particle information, animacy information, or word order information, while animacy information is not used in predicate processing. Hence, the PIAI model predicts that animacy information is used in argument processing before input of the predicate, but not in predicate processing.

To test this issue, four types of stimulus sentences were used: reversible canonical sentences, reversible scrambled sentences, non-reversible canonical sentences, and non-reversible scrambled sentences [see (1)–(4) below]. These were categorized into four conditions, as follows:

1. Reversible canonical sentence
  Kinoo sono-dansei-ga sono-otokonoko-o home-ta-rashii.
  Yesterday the man-NOM the boy-ACC praise-PAST-AUX.
  Yesterday, the man seemed to praise the boy.
2. Reversible scrambled sentence
  Kinoo sono-otokonoko-o sono-dansei-ga home-ta-rashii.
  Yesterday the boy-ACC the man-NOM praise-PAST-AUX.
  Yesterday, the man seemed to praise the boy.
3. Non-reversible canonical sentence
  Senshuu sono-onnanoko-ga sono-hon-o yon-da-souda.
  Last week the girl-NOM the book-ACC read-PAST-AUX
  Last week, the girl seemed to read the book.
4. Non-reversible scrambled sentence
  Senshuu sono-hon-o sono-onnanoko-ga yon-da-souda.
  Last week the book-ACC the girl-NOM read-PAST-AUX
  Last week, the girl seemed to read the book.

Then, in order to first test whether animacy information is used in argument processing before input of the predicate, we compared the reading times of the second argument between the reversible scrambled and non-reversible scrambled sentences. In Japanese, arguments can be interchanged. For example, in the sentence with canonical word order *Taroo-ga Pan-o Tabe-ta* (Taroo ate bread), these two arguments (Taroo-ga and Pan-o) are interchangeable without a change in meaning. By using this phenomenon, we can directly compare the reading times of the second argument (e.g., *Taroo-ga*) between the reversible scrambled sentences (e.g., *Pan-o Taroo-ga Tabeta*) and the non-reversible scrambled sentences (e.g., *Hanako-o Taroo-ga Hometa*) because of the common lexical item (i.e., *Taroo-ga*). If the animacy information of the first argument is used to reduce the possible thematic role assignment patterns of the second argument in real-time sentence comprehension, the second argument in the scrambled non-reversible sentence would be easier to comprehend than that in the scrambled reversible sentence.

Secondly, in order to test whether animacy information is used in predicate processing, we examined the interaction between the reversibility factor (i.e., reversible vs. non-reversible) and the scrambling factor (i.e., canonical vs. scrambled) among the reversible canonical, non-reversible canonical, reversible scrambled, and non-reversible scrambled sentences. Since the verbs are basically different between reversible and non-reversible sentences, it is hard to directly compare the reading times of the verb/predicate between these two types of sentences. Hence, to control the difference of verbs/predicates between the two, we used a 2×2 design with the scrambling factor and reversibility factor, and tested their interaction. Basically, scrambled sentences are harder to process than the corresponding canonical sentences in the Japanese language [2], [5], [20]. If the animacy information of arguments is used in predicate processing, the scrambling effect between the canonical non-reversible and scrambled reversible sentences would be larger than that between the canonical reversible and scrambled reversible sentences; in other words, animacy information would reduce the possible thematic role assignment patterns so that predicates in non-reversible sentences are easier to comprehend.

## Methods

Twenty-three Japanese native speakers (two female; mean age: 21.1, SD = 0.8) participated in this experiment. Written informed consent was obtained from each subject in accordance with the guidelines approved by the Tohoku University School of Medicine and the Helsinki Declaration of Human Rights, 1975.

Each target sentence consisted of two arguments (a subject marked by a nominative case particle and an object marked by an accusative case particle) and a verb followed by an auxiliary verb such as *souda, youda*, and *rashii*, which represent the meaning of a guess. Also, all sentences started with an adverb representing time such as kinoo (yesterday). All of the animate nouns were *sono-dansei*, *sono-josei*, *sono-otokonoko*, and *sono-onnanoko* (that man, that woman, that boy, and that girl, respectively), and these items were counter-balanced across all conditions [21]. Inanimate nouns in non-reversible sentences were like *hako* (box), *tsukue* (desk), *teeburu* (table), *isu* (chair), *kabe* (wall), and so on, which are high-frequency words in the Japanese language. Based on the above items, four types of stimulus sentences were created: reversible canonical sentences, reversible scrambled sentences, non-reversible canonical sentences, and non-reversible scrambled sentences (see (1)–(4) above).

Thirty-two items were prepared as target stimuli for this experiment. Additionally, fifty-four filler items were prepared, which were not included in the data analysis. The filler items comprised transitive sentences, scrambled sentences, sentences including a relative clause, passive sentences, and scrambled passive sentences. The words used were similar to those of the target sentences.

The experiment was conducted with a Windows-based notebook computer running E-prime 2.0 (PST Inc., Pittsburgh, PA) software. Participants were timed in a phrase-by-phrase (a phrase is defined as a noun with a case particle, a verb, an auxiliary verb, or adverb in this experiment), self-paced, non-cumulative moving-window reading task [22]. Stimuli initially appeared as dots with intervening spaces indicating the segments, and participants pressed the enter key to reveal each subsequent region of the sentence, in turn causing the present region to revert to dots. At the end of each sentence, a yes/no question appeared on a new screen. All sentences fit on a single line and were presented without line breaks. Participants answered by pressing either the y or n key. No feedback was provided. Before this experiment, participants read written instructions and completed eight practice trials. The experiment took the participants approximately 30 minutes.

## Results

Here, in order to easily identify examples of each condition, we would like to show an example for each condition again. Also, we added “/” to show how to present stimuli to participants by using phrase-by-phrase manner.

1. Reversible canonical sentence
  Kinoo/sono-dansei-ga/sono-otokonoko-o/home-ta-rashii.
  Yesterday the man-NOM the boy-ACC praise-PAST-AUX.
  Yesterday, the man seemed to praise the boy.
2. Reversible scrambled sentence
  Kinoo/sono-otokonoko-o/sono-dansei-ga/home-ta-rashii.
  Yesterday the boy-ACC the man-NOM praise-PAST-AUX.
  Yesterday, the man seemed to praise the boy.
3. Non-reversible canonical sentence
  Senshuu/sono-onnanoko-ga/sono-hon-o/yon-da-souda.
  Last week the girl-NOM the book-ACC read-PAST-AUX
  Last week, the girl seemed to read the book.
4. Non-reversible scrambled sentence
  Senshuu/sono-hon-o/sono-onnanoko-ga/yon-da-souda.
  Last week the book-ACC the girl-NOM read-PAST-AUX
  Last week, the girl seemed to hit the woman.

Regarding the accuracy rates of probe questions, ANOVA showed the statistical significance of all main effects of scrambling (F(1,22) = 22.02, p<0.002) and reversibility (F(1,22) = 23.48, p<0.001), and their interaction (F(1,22) = 4.84, p<0.05). Overall, scrambled sentences were harder to comprehend than the corresponding canonical ones, and reversible sentences were harder to comprehend than the corresponding non-reversible ones. These results are consistent with previous findings [5], [6], [9], [14].

Before performing the analysis of reading times, reaction times outside of 3.5 standard deviations (SD) at both the high and low ranges were replaced to the maximum or minimum by boundaries indicated by 3.5 SD from the individual means of participants in each category, according to previous studies [5], [6]. This procedure affected less than 2% of all data. The statistical tests analyzed both subject (F1) and item (F2) variability. Only stimulus items for which participants provided correct responses were used in the analyses of reading times. The accuracy rates and reading times described above are shown in Table 1. Based on our experimental hypotheses, we conducted two statistical tests.

**Table 1:** Results.

Accuracy rates of probe question (%)		
	Mean	SD
Reversible/Canonical	91.3	12.7
Reversible/Scrambled	77.71	22.2
Non-reversible/Canonical	99.45	2.6
Non-reversible/Scrambled	95.65	1.8

Reading times for the second argument position (ms)		
	Mean	SD
Reversible	997	218
Non-reversible	1189	216

Reading times for the verb position (ms)		
	Mean	SD
Reversible/Canonical	899	268
Reversible/Scrambled	998	335
Non-reversible/Canonical	626	151
Non-reversible/Scrambled	719	242

**Figure 1:**
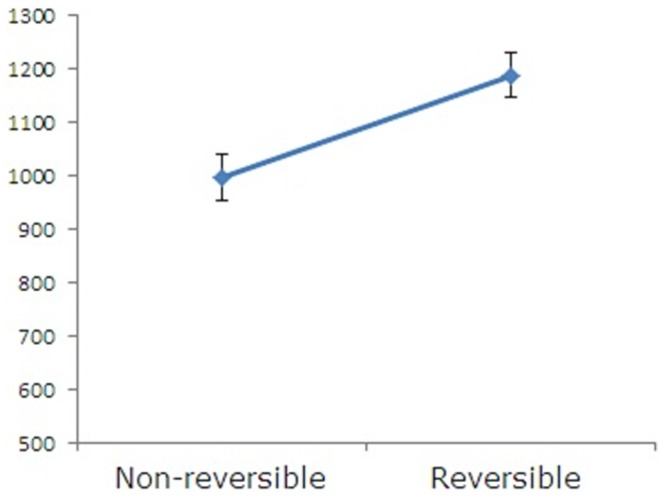
Reading times at the second argument position. Mean reading times for the second argument between the reversible scrambled sentence and the non-reversible scrambled sentence conditions. Error bar denotes standard mean error.

The first is an ANOVA of reading times of the second argument between the reversible scrambled and the non-reversible scrambled sentences. Our results showed that the reading times of the second argument for the non-reversible scrambled sentences were shorter than those for the reversible scrambled sentences (F1(1,22) = 14.79, p<0.001, F2(1,31) = 9.93, p<0.05). This indicates that the animacy information of the first argument is used to reduce the possible thematic role assignment patterns of the second argument in real-time sentence comprehension, as the PIAI model predicted. This data is shown in Fig. 1.

**Figure 2:**
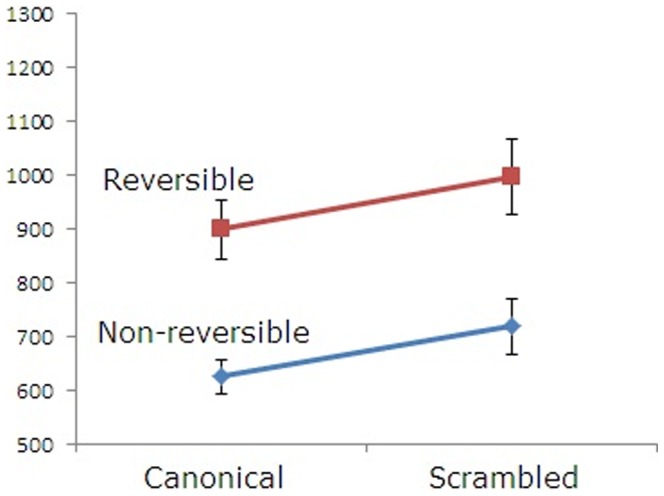
Reading times at the verb position. Mean reading times for the predicate among four conditions: reversible canonical, non-reversible canonical, reversible scrambled, and non-reversible scrambled sentences. Error bar denotes standard mean error.

The second is a 2×2 ANOVA of reading times at the predicate position with the reversibility factor and the scrambling factor among the reversible canonical, non-reversible canonical, reversible scrambled, and non-reversible scrambled sentences. In our results, the main effects of both the reversibility and scrambling factors were statistically significant (reversibility factor: F1(1,22) = 43.20, p<0.001; F2(1,31) = 20.96, p<0.005; scrambling factor: F1(1,22) = 6.80, p<0.05; F2(1,31) = 22.96, p<0.005). In contrast, there was no statistical significance in their interaction (F1(1,22) = 0.17, p = 0.89, F2(1,31) = 0.28, p = 0.61), in which reversible sentences showed no different scrambling effect from the corresponding non-reversible sentences. This indicates that the animacy information of arguments is not used in predicate processing. This data is shown in Fig. 2.

Note that we do not show any other statistical results of reading times at other positions, since they are not related to the purpose of the present study. To be more specific, at the first argument position, there is no concrete purpose and hypothesis to directly compare reading times among four conditions.

## Discussion

The purpose of the current study was to examine whether animacy information is used in real-time sentence comprehension in the Japanese language or not. To this end, by using a self-paced reading task, we first tested whether animacy information is used at the second argument position during real-time sentence comprehension or not, and, secondly, whether animacy information is used at the predicate position during real-time sentence comprehension or not. Our results indicate that the animacy information of the first argument is used to reduce the possible thematic role assignment patterns of the second argument in real-time sentence comprehension, as the PIAI model predicted. Regarding the second hypothesis, our results do not provide any evidence to support the hypothesis that the animacy information of arguments is used in predicate processing, as the PIAI model also predicted. First, we would like to discuss the interpretation of our results.

Regarding the first test that animacy information is used at the second argument position during real-time sentence comprehension, because of the same lexical item (i.e., *Taroo-ga*), we can directly compare the reading times of the second argument (e.g., *Taroo-ga*) between the reversible scrambled sentences (e.g., *Pan-wo Taroo-ga Tabeta*) and the non-reversible scrambled sentences (e.g., *Hanako-wo Taroo-ga Hometa*). Between these two conditions, the differences are found in their first argument (e.g., *Pan-wo* and *Hanako-wo*) and their predicate (e.g., *Tabeta* and *Hometa*). At the second argument position, their predicates are not presented, since Japanese is a SOV language which has a predicate at the end of a clause or sentence. Since we observed a statistically significant difference in the reading times of the second argument between the two conditions, even when the same lexical item was presented, this difference should be caused by the difference of the first argument. Since the reading time of the second argument in non-reversible scrambled sentences was shorter than that in reversible scrambled sentences, the results strongly support the hypothesis that the animacy information of the first argument reduces the possible thematic role assignment patterns of the second argument, indicating that animacy information is used at the second argument position during real-time sentence comprehension. The above result is consistent with previous findings that showed reversible sentences are harder to understand [13]–[17].

Regarding the second test that animacy information is not used at the predicate position during real-time sentence comprehension, verbs are basically different between reversible and non-reversible sentences. Hence, to control the difference of verbs/predicates between the two conditions, we used a 2×2 design with a reversibility factor and a scrambling factor, and tested their interaction. Consistent with previous findings that scrambled sentences are harder to process than the corresponding canonical sentences in Japanese [2], [5], [20], we found longer reading times of predicates in the scrambled sentence condition than in the canonical sentence condition. Also, we found longer reading times of predicates in the reversible sentence condition than in the non-reversible sentence condition, but these results are relatively meaningless with respect to the second hypothesis, since compared lexical items themselves differ between the two conditions, as described in the Introduction. To this end, we tested the interaction between the scrambling and reversibility factors, and we did not find any statistical significance. Since we did find statistically significant main effects for both the reversibility and scrambling factors, it may be impossible to explain that the non-statistical significance found in the interaction effect is due to the small number of subjects or to less statistical power. Hence, our results suggest that animacy information is not used at the predicate position during real-time sentence comprehension.

Next, we would like to discuss our results in the context of the Japanese simplex sentence comprehension models. As described above, both of the experimental results reported here support the predictions of the PIAI model, but not those of Chujo's model. The PIAI model predicts that the animacy information of arguments is used in the processing of the argument before input of the predicate. Actually, our results showed that, in the second argument position, the non-reversible sentence condition is easier to comprehend than the reversible sentence condition. This supports the PIAI model prediction that the animacy information of the first argument is used to reduce the possible thematic role assignment patterns of the second argument in real-time sentence comprehension, so that non-reversible sentences with fewer possible thematic patterns are easier to understand than reversible sentences with more possible thematic patterns. In addition, we found the reversibility factor had no effect on the processing of the predicate, suggesting that during the latter, the integration/completion of sentence comprehension does not require or use the animacy information of arguments. In the PIAI model, since the animacy information in arguments has not been assumed to be used in the processing of the predicate, our results support its prediction as well.

However, there is at least one remaining issue with regard to the use of animacy information in sentence comprehension: whether the animacy information of the first argument is used for the processing of the first argument or not. While our results indicate that the animacy information of the first argument is used to interpret the second argument, our experiment cannot examine whether the animacy information of the first argument is used for the processing of the first argument or not. The reason is that in our experiment, the accusative-marked argument (i.e., *o*-marked argument) differs between the reversible and non-reversible sentence conditions in terms of the lexical item (e.g., animate nouns in the reversible sentences, but inanimate nouns in the non-reversible sentences). This means that we cannot directly compare the reading times between the two. Since, in order to test this issue, we have to control the lexical items, it is necessary to conduct another experiment.

## Conclusions

The purpose of the current study was to examine whether animacy information is used in real-time sentence comprehension in the Japanese language, which is one of SOV languages, or not. To this end, by using a self-paced reading task, we tested whether animacy information is used in the second argument position during real-time sentence comprehension or not, and whether animacy information is used in the predicate position during real-time sentence comprehension or not. Our results support the PIAI model predictions but not Chujo's model predictions. In order to comprehend a sentence in SOV languages, readers/listeners cannot use information from a predicate at the beginning of sentence comprehension, which differs from the case in SVO languages. To cover all of the human sentence comprehension mechanisms, it is necessary to examine which points differ between SVO and SOV languages, and which points are the same between the two.
